# Supplementary material for: splitSMLM, a spectral demixing method for high-precision multi-color localization microscopy applied to nuclear pore complexes
Source: Commun Biol. 2022 Oct 17;5:1100. doi: 10.1038/s42003-022-04040-1 (PMC9576791; doi:10.1038/s42003-022-04040-1)
Supplement: Supplementary file 2 — Supplementary Material [file 42003_2022_4040_MOESM2_ESM.pdf]

A spectral demixing method for high-precision multi-color localization microscopy applied to nuclear pore complexes

Leonid Andronov, Rachel Genthial, Didier Hentsch, Bruno P. Klaholz

Supplementary Figures & Software Installation Instructions

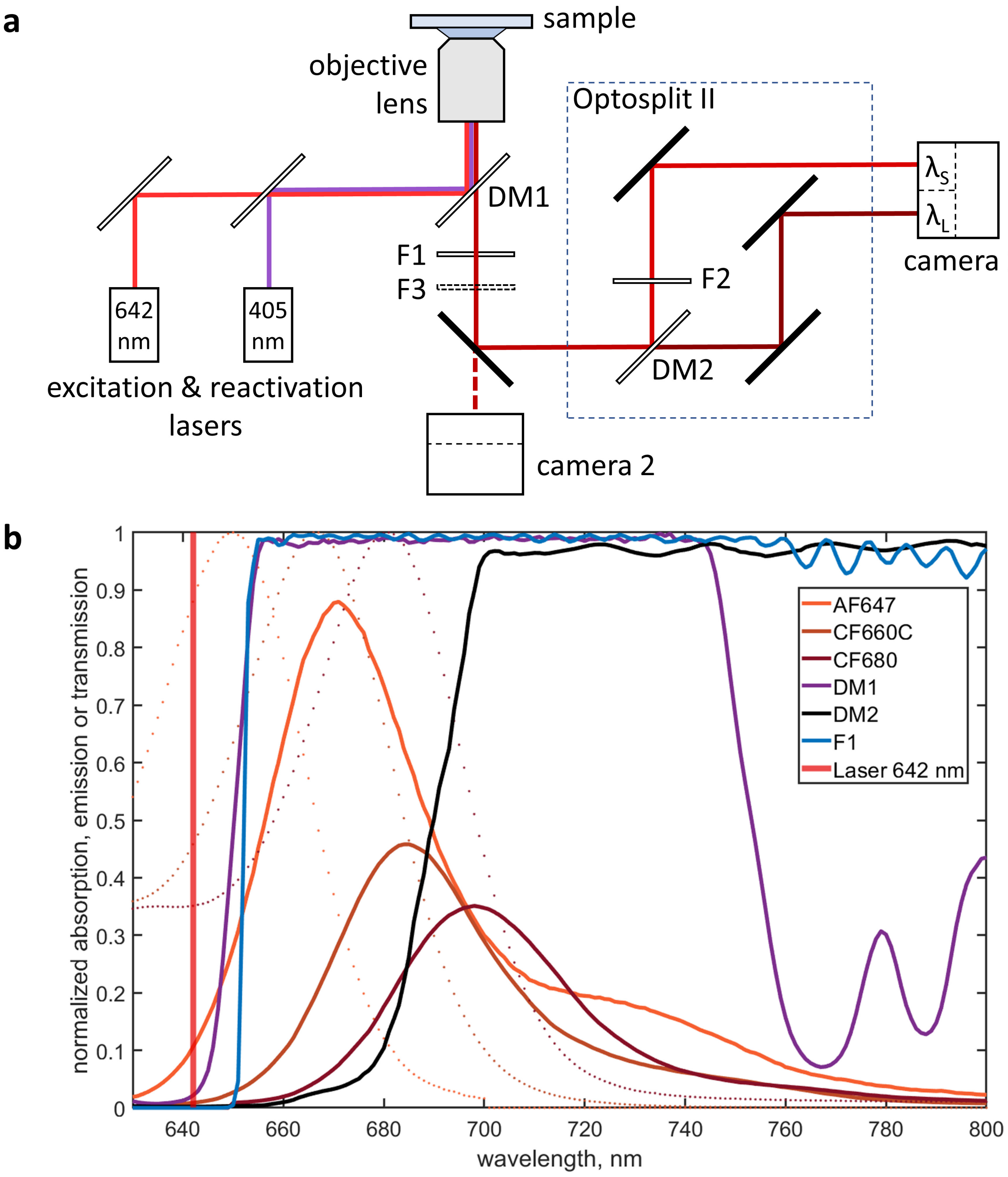

Suppl. Fig. S1 Optical scheme and spectra

(**A**) Optical scheme of the splitSMLM system. (**B**) Spectra of fluorophores and filters used in this study. The emission spectra of the fluorophores are normalized with respect to their excitation intensity at the laser wavelenght.

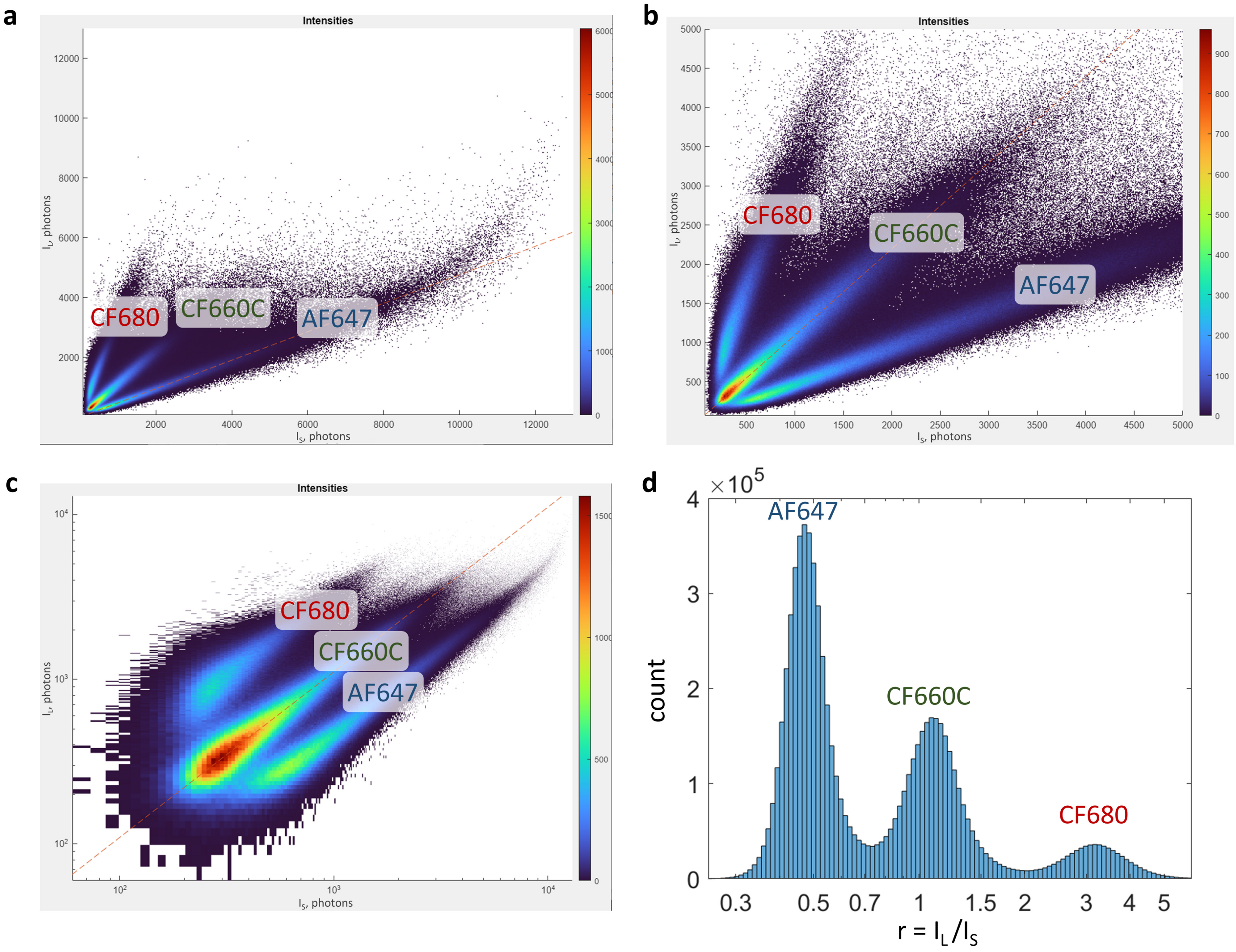

Suppl. Fig. S2 Various representations of splitSMLM data

(**A-C**) Bivariate histograms of intensities in the λ_L and λ_S channels, showing the full extent of the intensities (A), only the localizations with less than 5000 photons (B) and full extent of the data on a log-log plot (C). (**D**) Univariate histogram of ratios *r* on a semi-log plot with peaks corresponding to different fluorophore species.

		AF647	spectral difference ↔	CF660C	spectral difference ↔	CF680	spectral difference ↔	AF647
	Average emission wavelength	686.31 nm		692.60 nm		702.82 nm		
	No image splitter		6.29 nm		10.22 nm		16.51 nm	
	Average emission in λ_S	671.78 nm		677.95 nm		682.93 nm		
	Average emission in λ_L	711.63 nm		707.28 nm		710.36 nm		
Demixed coordinates	All from λ_S		6.17 nm		4.98 nm		11.16 nm	
Demixed coordinates	All from λ_L		4.36 nm		3.09 nm		1.27 nm	
Demixed coordinates	From brightest channel		35.50 nm		32.41 nm		38.58 nm	
Demixed coordinates	Weighted mean		6.27 nm		10.17 nm		16.44 nm	
Demixed coordinates	Simple mean		0.91 nm		4.03 nm		4.94 nm	
	Theoretical $r = I_L / I_S$	0.57		0.99		2.59		
	Experimental $r = I_L / I_S$	0.48		1.11		3.23		

Suppl. Fig. S3 Spectral properties of fluorophores within the λ_S and λ_L channels of image splitter and within demixed data

The spectral difference denotes the difference between the average emission wavelengths of the corresponding fluorophore pairs. The blue line indicates similar spectral difference between the fluorophores without image splitter and after demixing with the "weighted mean" method, the slight difference originates from F2 filter inside the image splitter.

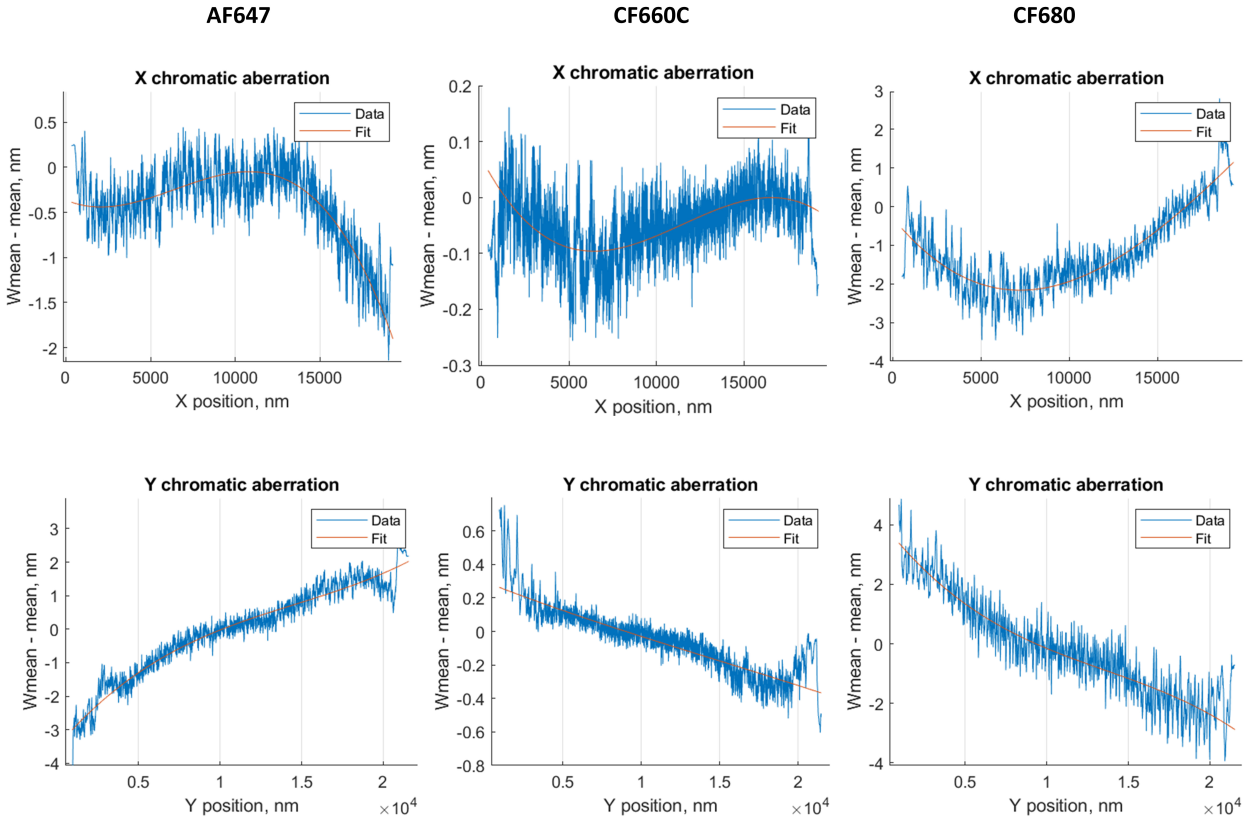

Suppl. Fig. S4 Correction of chromatic aberrations in SplitViSu

The graphs show residual chromatic shift between the coordinates of the fluorophores, calculated as the "weighted mean" and the "simple mean" of the input coordinates (blue, using a moving average window to reduce fluctuations). The polynomial fit of this shift (red curves) is then subtracted from the "weighted mean" coordinates that provides correction of the residual chromatic error.

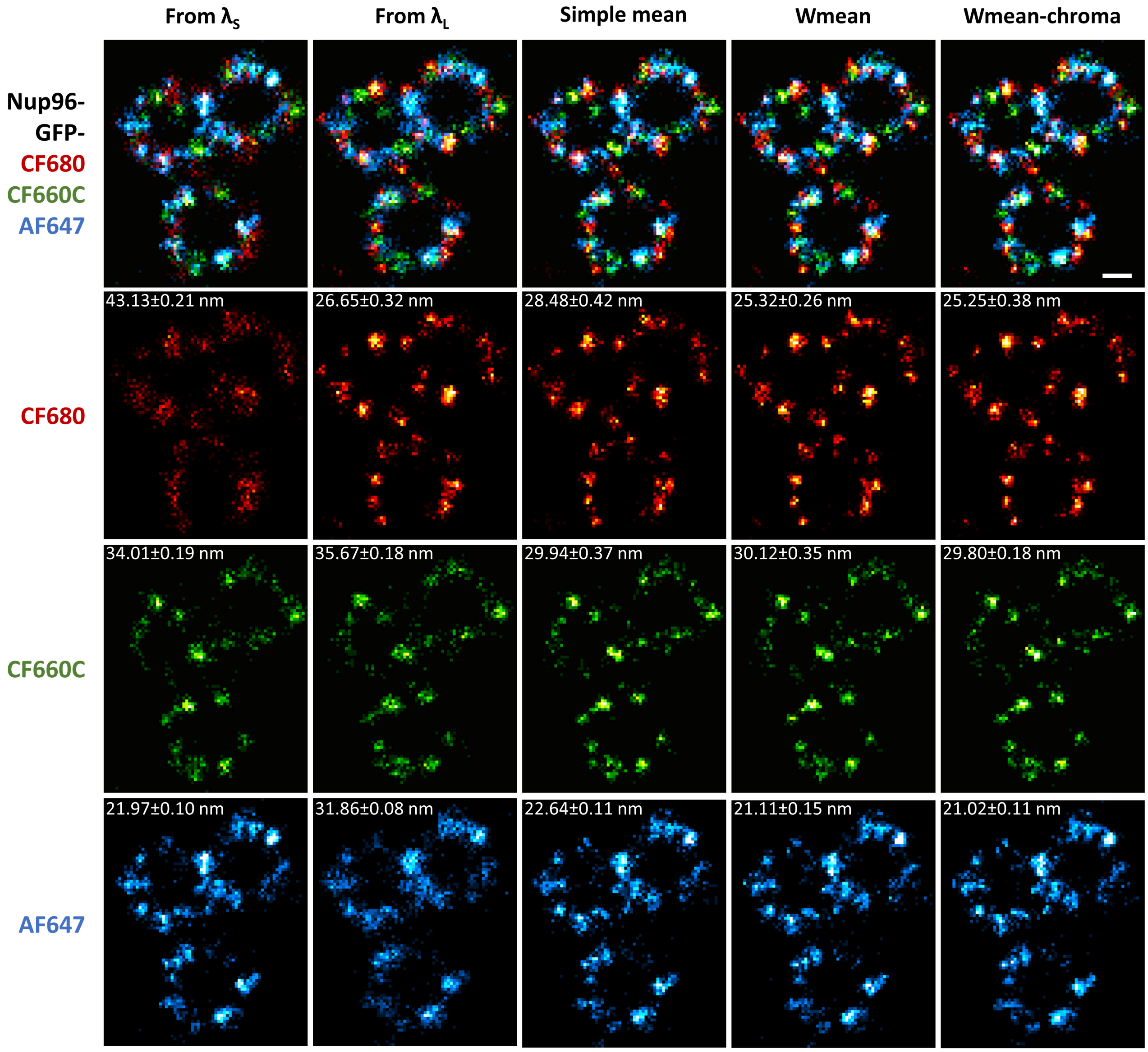

Suppl. Fig. S5 Positive control for chromatic error correction

Images of Nup96, simultaneously labelled with three different secondary antibodies, after demixing in SplitViSu using corresponding methods for calculation of output coordinates. The resolution value, calculated according to the $FRC_{1/7}$ criterion from images of a whole bottom part of the nuclear envelope is indicated on top of every image. Scale bar, 50 nm.

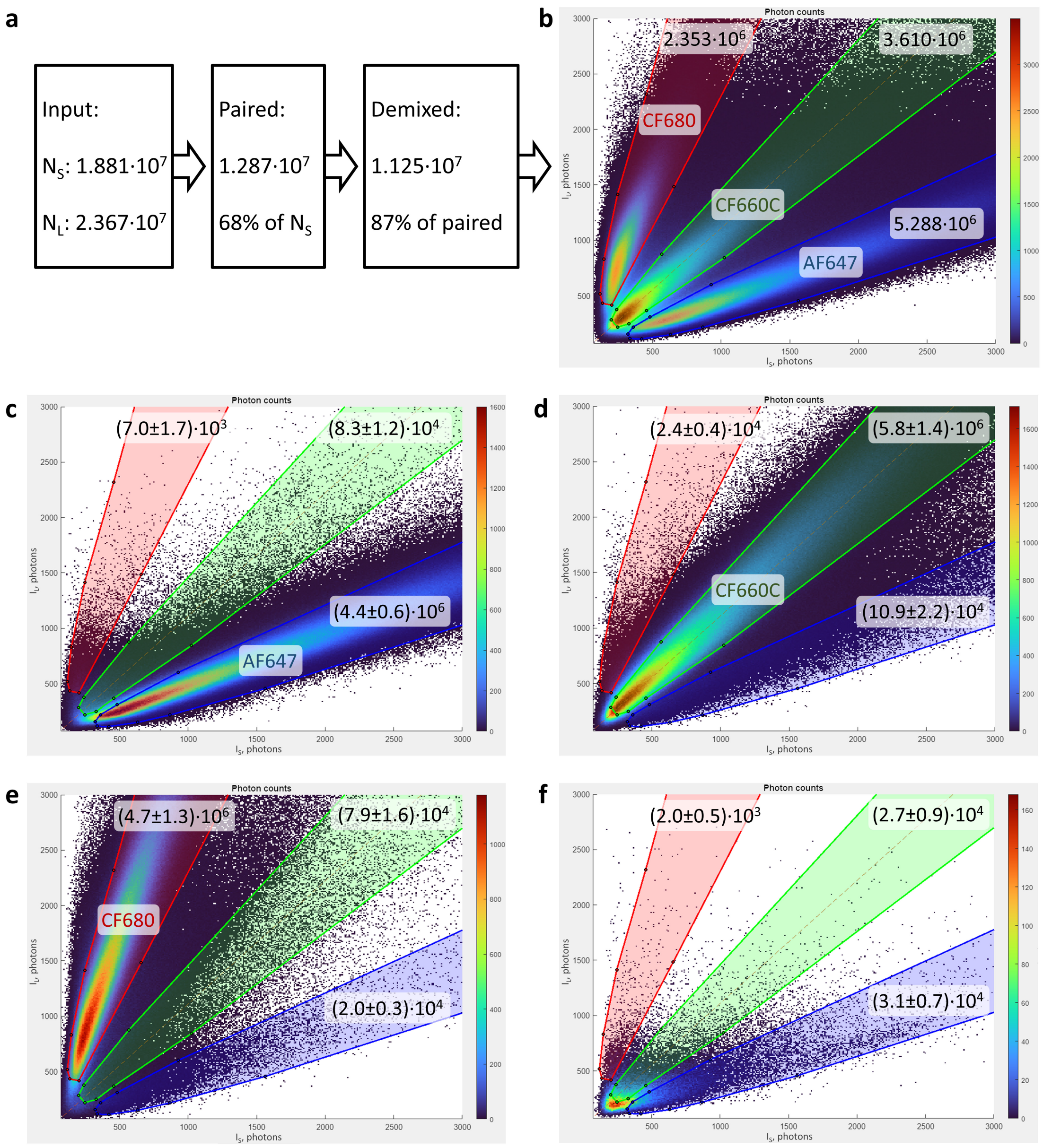

Suppl. Fig. S6 Cross-talk and rejection of localizations in splitSMLM

(**A-B**) Example of three-color demixing in SplitViSu. (**A**) Number of input localizations within the λ_S and λ_L channels (N_S and N_L, correspondingly), number of localizations after pairing and number of demixed output localizations in all three channels (after selecting the sectors in the bivariate histogram shown in B). (**B**) Demixed localizations within each output channel using the appropriate sector regions. (**C-E**) Demixed localizations within each output channel using the same sector regions and single-labelled samples. (**F**) Demixed localizations within a non-labelled sample. The values show mean ± standard deviation of the number of localizations from 5 datasets for each condition.

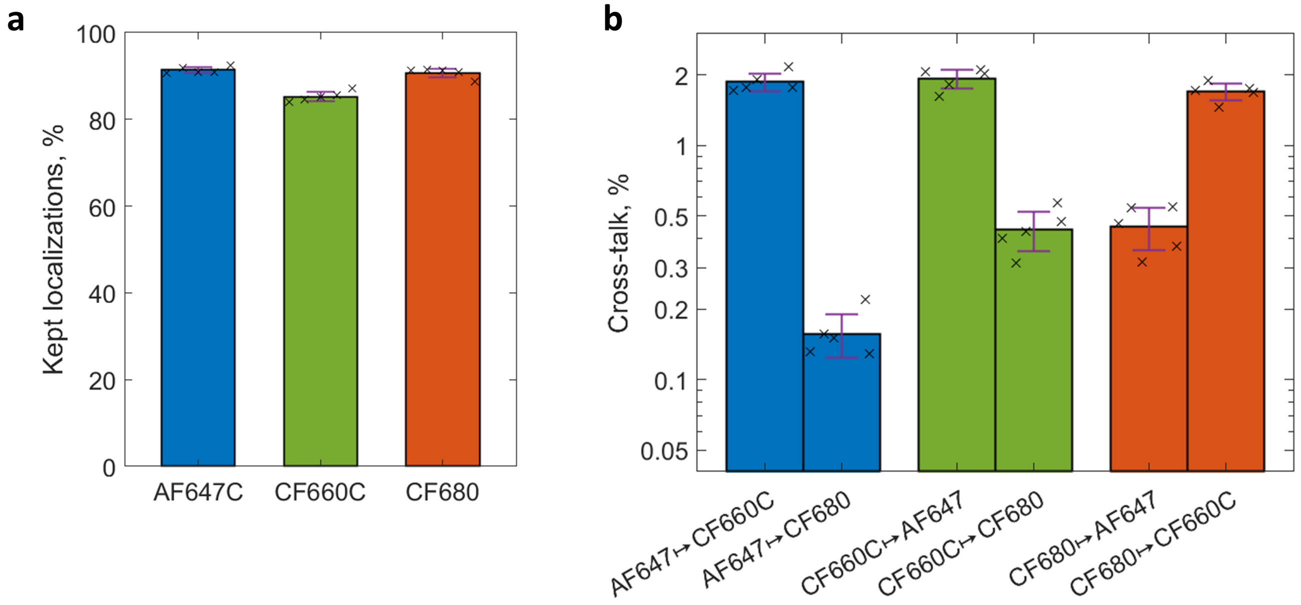

Suppl. Fig. S7 Rejection of localizations and cross-talk in splitSMLM

(**A**) Percentage of localizations found within the sector regions used for demixing. (**B**) Cross-talk for corresponding channel pairs. The values show mean ± standard deviation obtained from 5 single-labelled datasets for each condition.

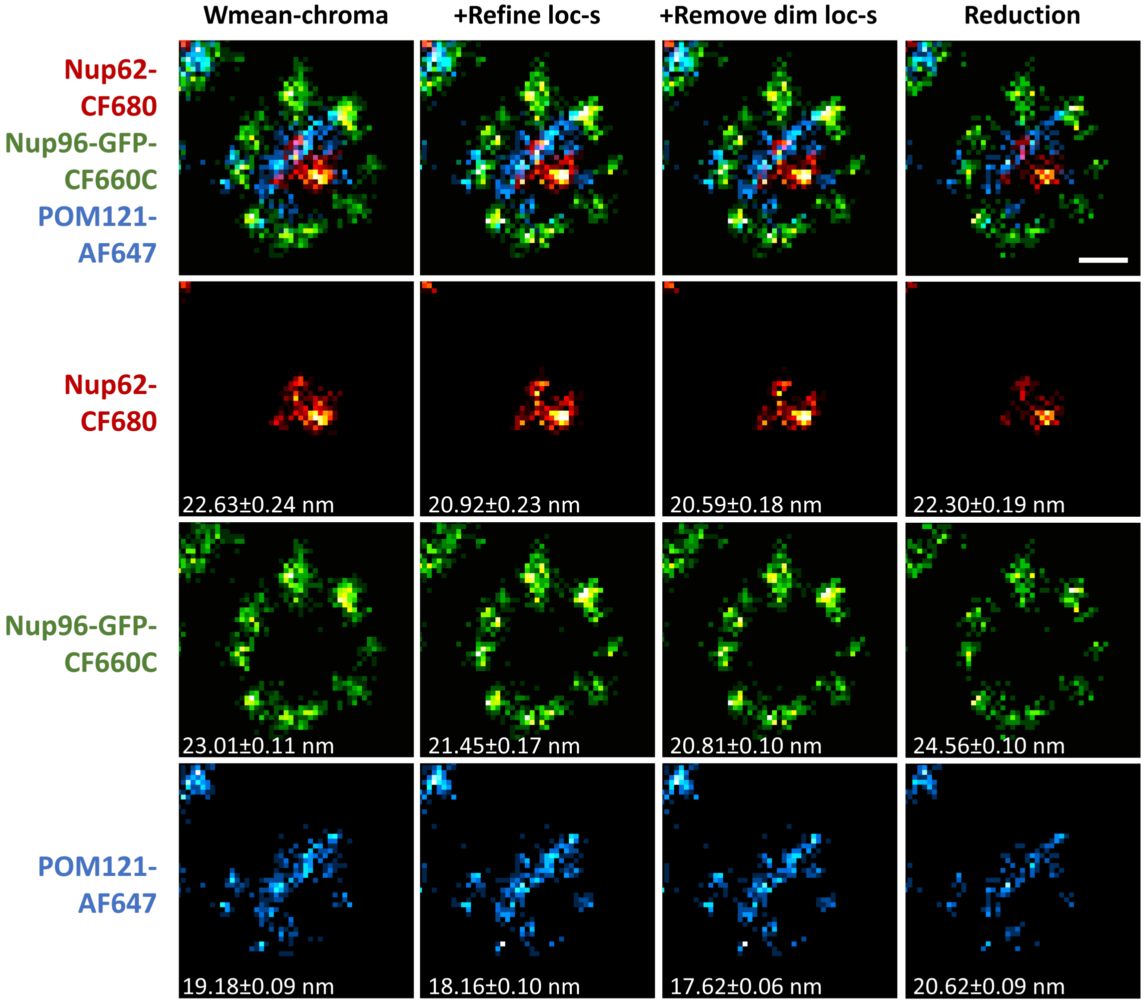

Suppl. Fig. S8 Refinement of multiple localizations

"Top views" of the NPC's in a U2OS cell with immunofluorescently labelled Nup62 (red), Nup96 (green) and Pom121 (blue). The refinement method used for the corresponding column is shown on top. $FRC_{1/7}$ resolution values were calculated from the images containing a whole bottom part of a nucleus (about 20x20μm^2) and are shown at the bottom. Scale bar, 50 nm.

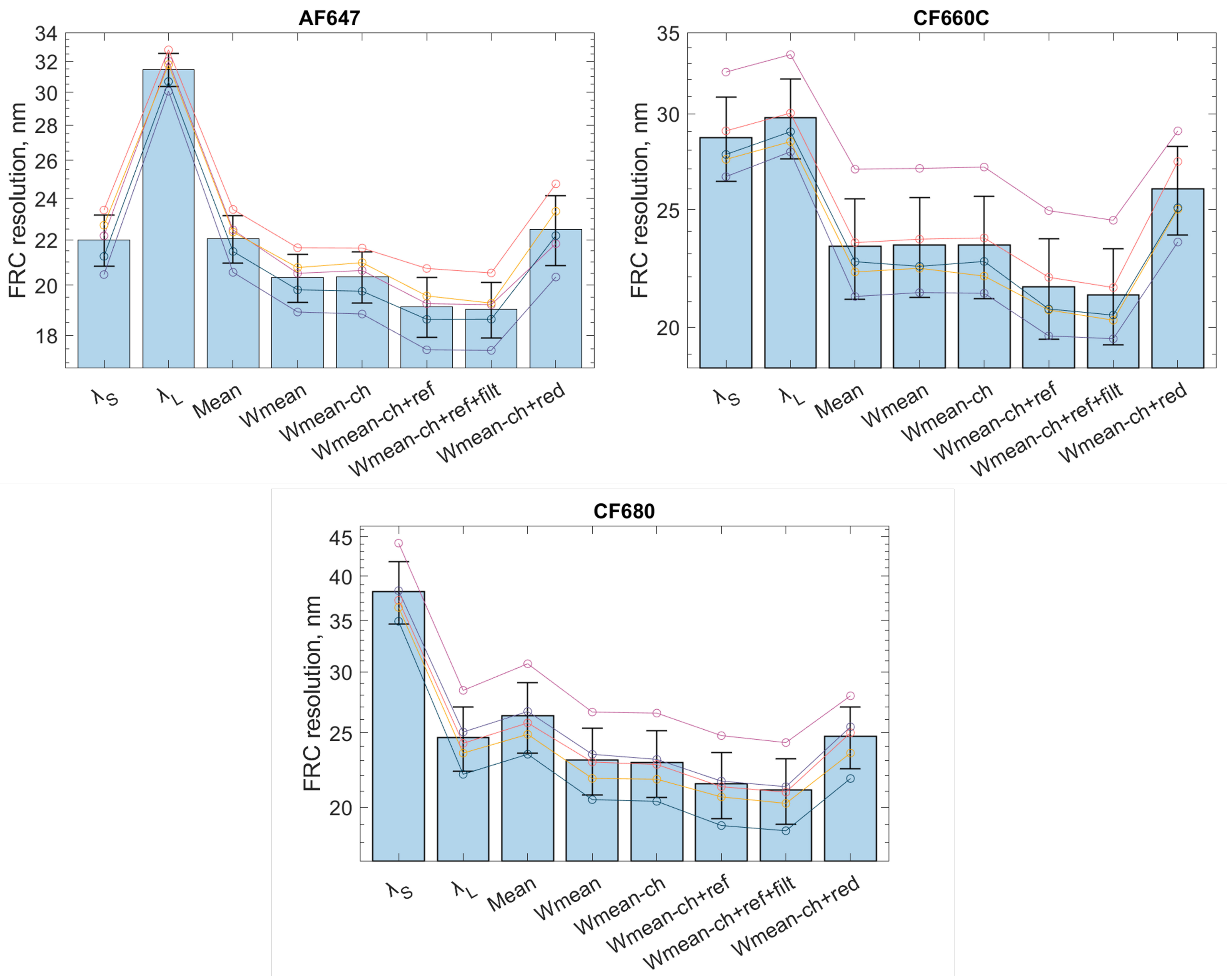

Suppl. Fig. S9 Resolution improvement after data processing

FRC$_{1/7}$ resolution of datasets processed with different methods for calculation of output coordinates: "λ_S" – the output equals to the input coordinates from the λ_S channel (X_S); "λ_L" – the output equals to the input coordinates from the λ_L channel (X_L); "Mean" – the output equals to the mean value of X_S and X_L; "Wmean" – the output equals to the intensity-weighted mean of X_S and X_L; "Wmean-ch" – the output equals to "Wmean" with subtraction of residual chromatic shifts; "Wmean-ch+ref" – the output equals to "Wmean-ch" with subsequent refinement of multiple localizations; "Wmean-ch+ref+filt" – the output equals to "Wmean-ch+ref" with subsequent removal of dim localizations; "Wmean-ch+ref" – the output equals to "Wmean-ch" with subsequent reduction of multiple localizations. The lines connect FRC$_{1/7}$ resolution values for same input datasets. The y-axis is in a logarithmic scale.

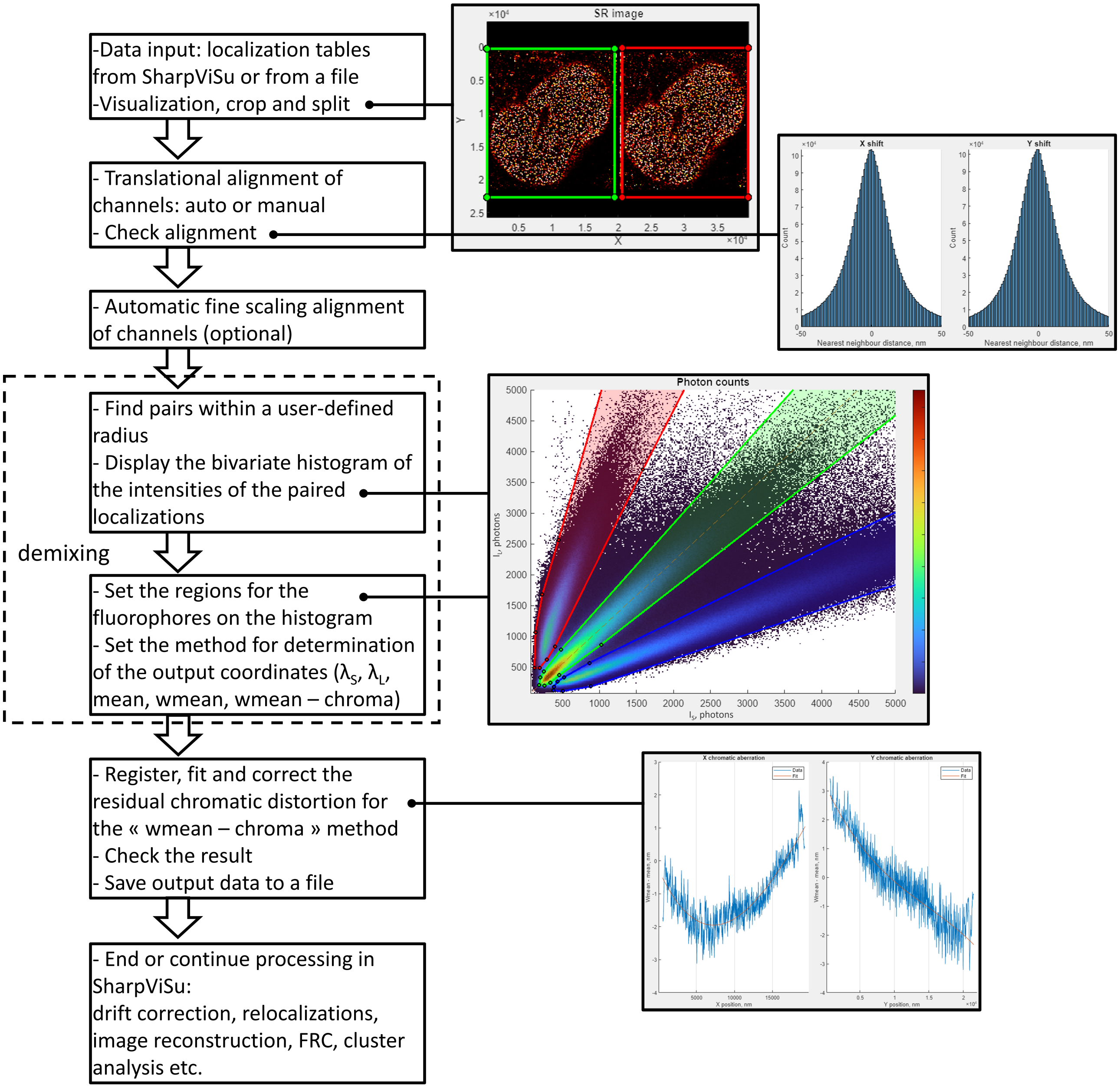

Suppl. Fig. S10 Workflow of fluorophore demixing in SplitViSu

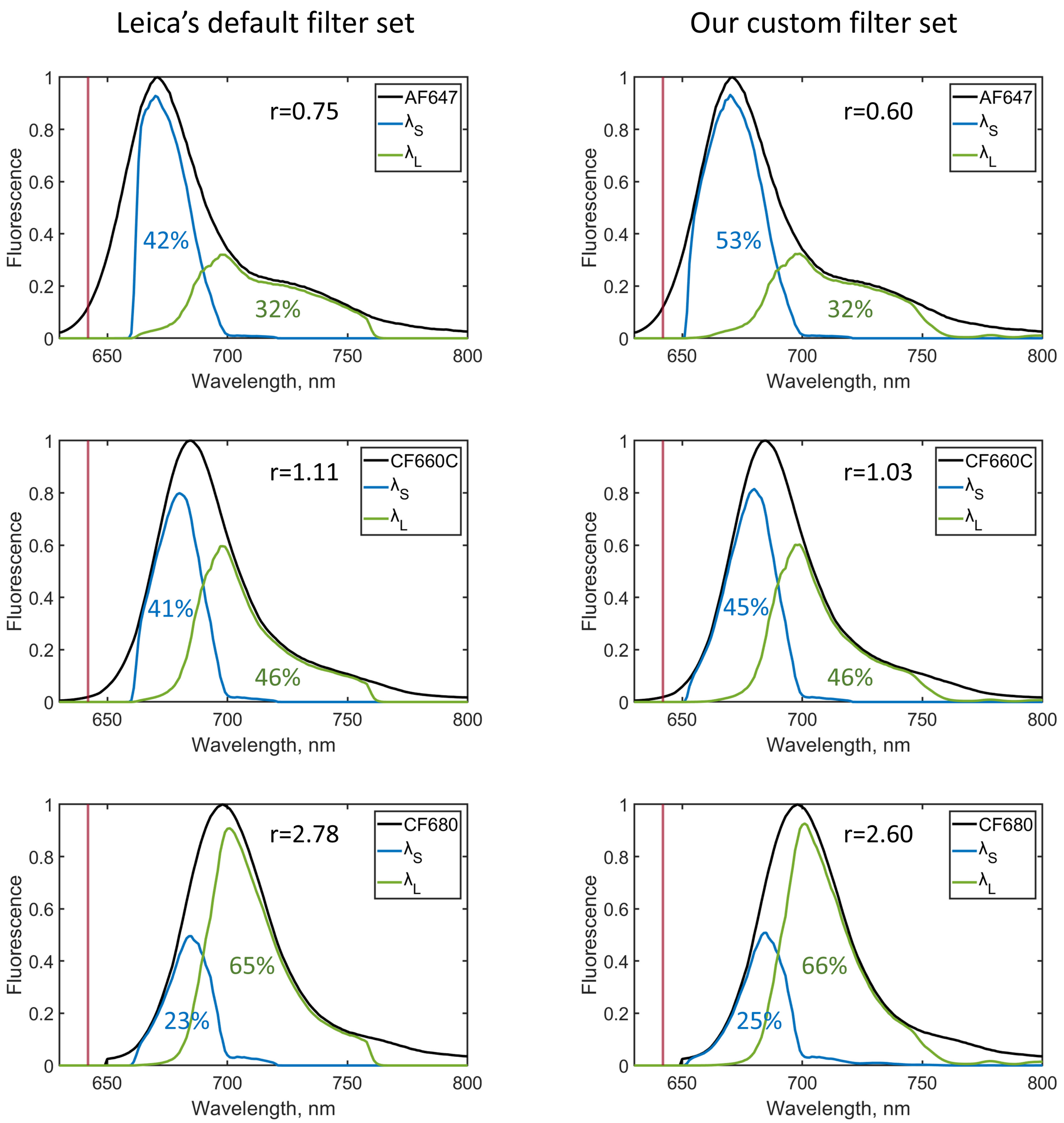

Suppl. Fig. S11 Improvement of photon collection and spectral discrimination with appropriate fluorescence filters

Calculated spectra within the λ_S (blue) and λ_L (green) channels of the image splitter using Leica's default filter set 642HP-T (ZT405/642rpc + et710_100lp + ET650LP, left column) and our custom filter set (FF545/650-Di01 + BLP01-532R + ZET635NF, right column) for AF647 (first row), CF660C (second row) and CF680 (third row). The percentage of total emission of the fluorophore that can be detected within the corresponding channel of the image splitter is indicated.

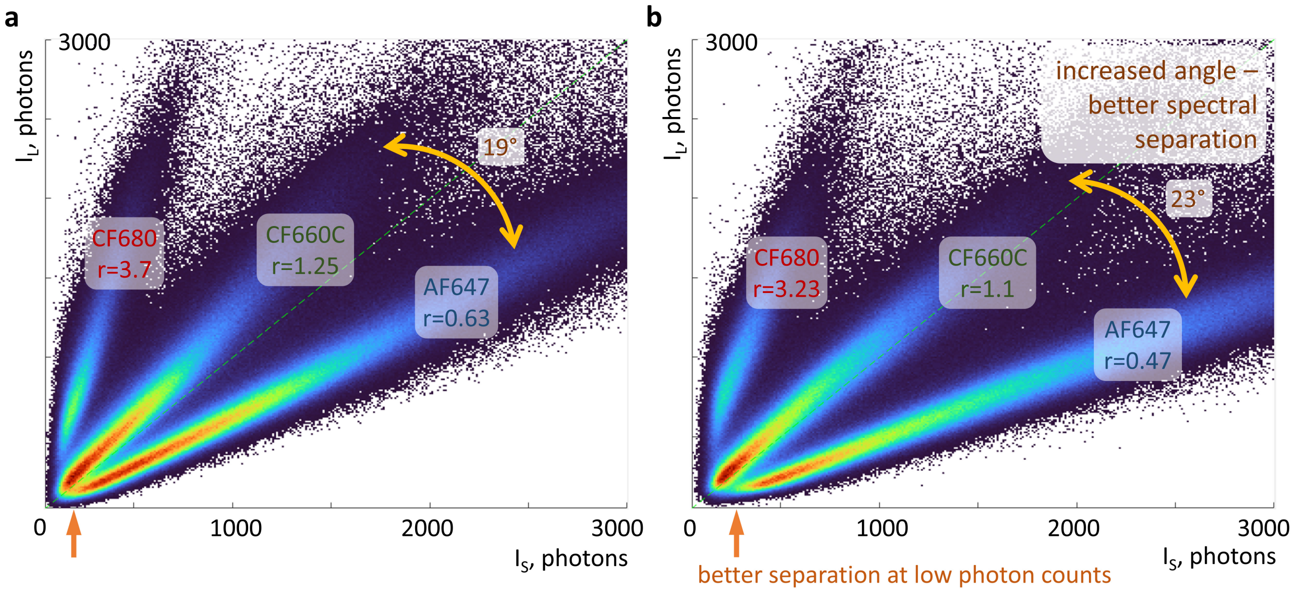

Suppl. Fig. S12 Improvement of the spectral separation of fluorophores with appropriate fluorescence filters

(**A**) Experimental bivariate histogram of photon counts of a triple-labelled sample using a standard filter set Leica GSD 642HP-T (ZT405/642rpc + et710_100lp + ET650LP). (**B**) Bivariate histogram of photon counts of the same sample using our custom filter set (FF545/650-Di01 + BLP01-532R + ZET635NF) allows better separation of dim fluorophores due to detection of fluorescence close to the laser line.

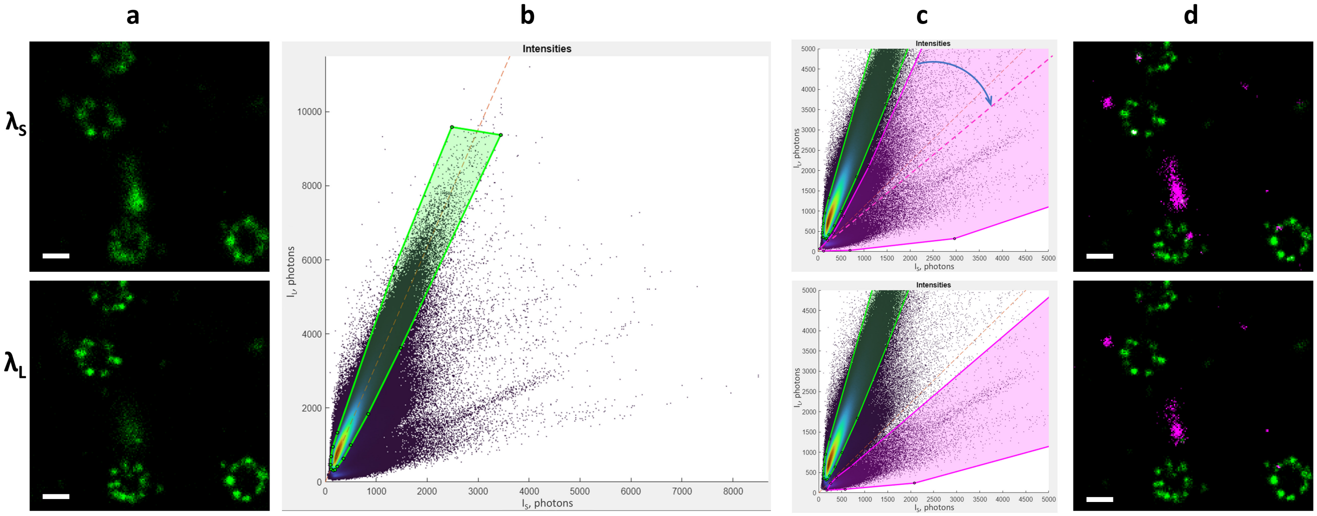

Suppl. Fig. S13 Removal of spurious localizations in splitSMLM

(**A**) SR images of a single-labelled sample Nup96-AF647, reconstructed from all localizations detected in the λ_S or λ_L channels. (**B**) Bivariate histogram of photon counts of this sample where the green sector corresponds to AF647. (**C**) Spectral demixing using a sector for AF647 (green) and two different sectors for spurious localizations (magenta). (**D**) Demixing in SplitViSu using the corresponding sectors in (C) allows separation of reliable signal (green) from spurious localizations (magenta). Scale bars, 100 nm.

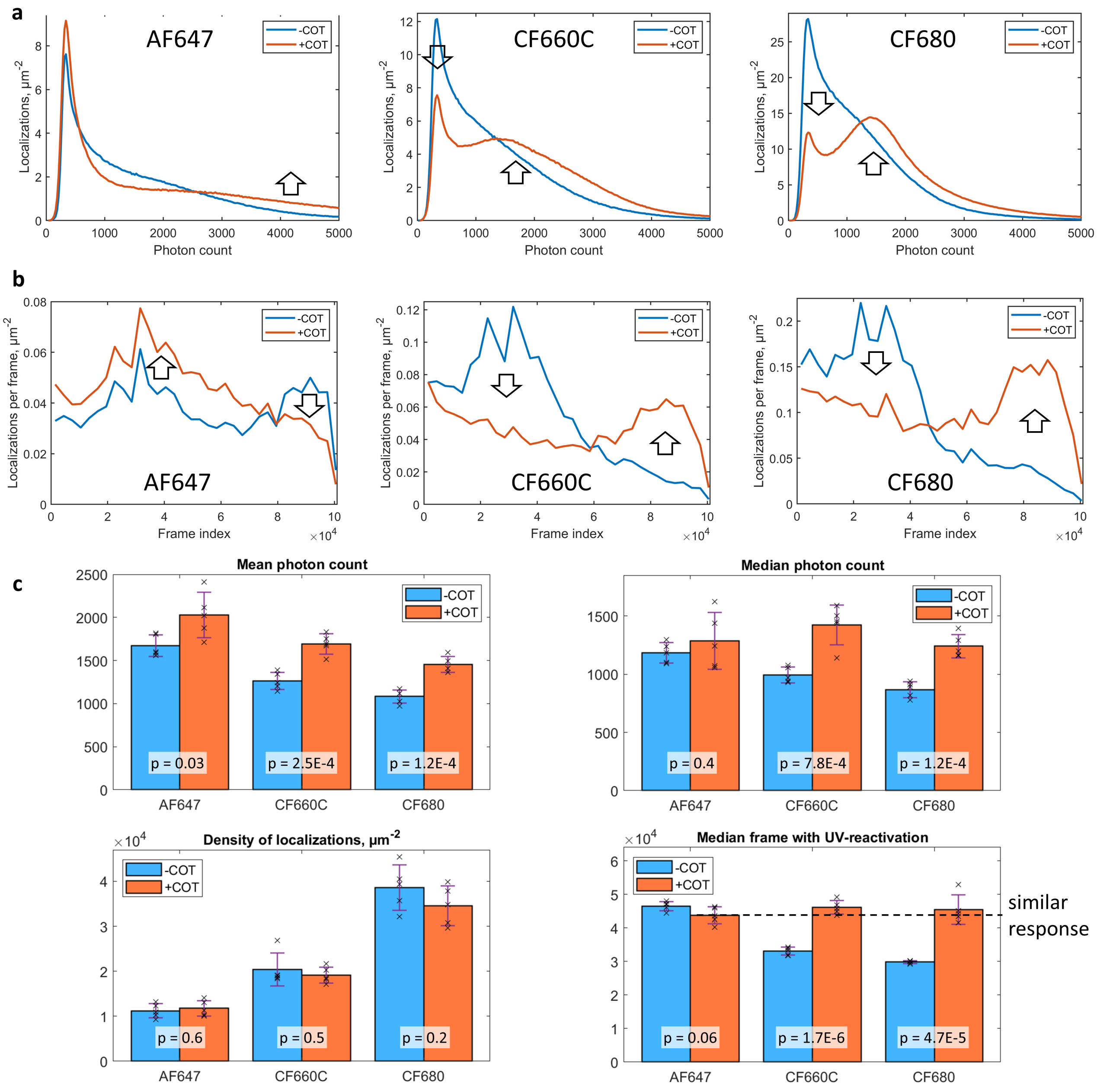

Suppl. Fig. S14 Behavior of fluorophores in a COT-supplemented imaging buffer

(**A**) Typical photon counts of fluorophores in a buffer with (red) or without (blue) COT. (**B**) Response on reactivation light – density of localizations per frame with stepwise increase of the 405 nm laser power in a buffer with (red) or without (blue) COT. (**C**) Statistics of fluorophore behavior demonstrates a significant increase in the photon count in the COT-supplemented buffer for all three fluorophores, while preserving the density of localizations and therefore equalizing the fluorophore response on reactivation with 405 nm light. (**A)** & (**B)** are individual representative datasets.

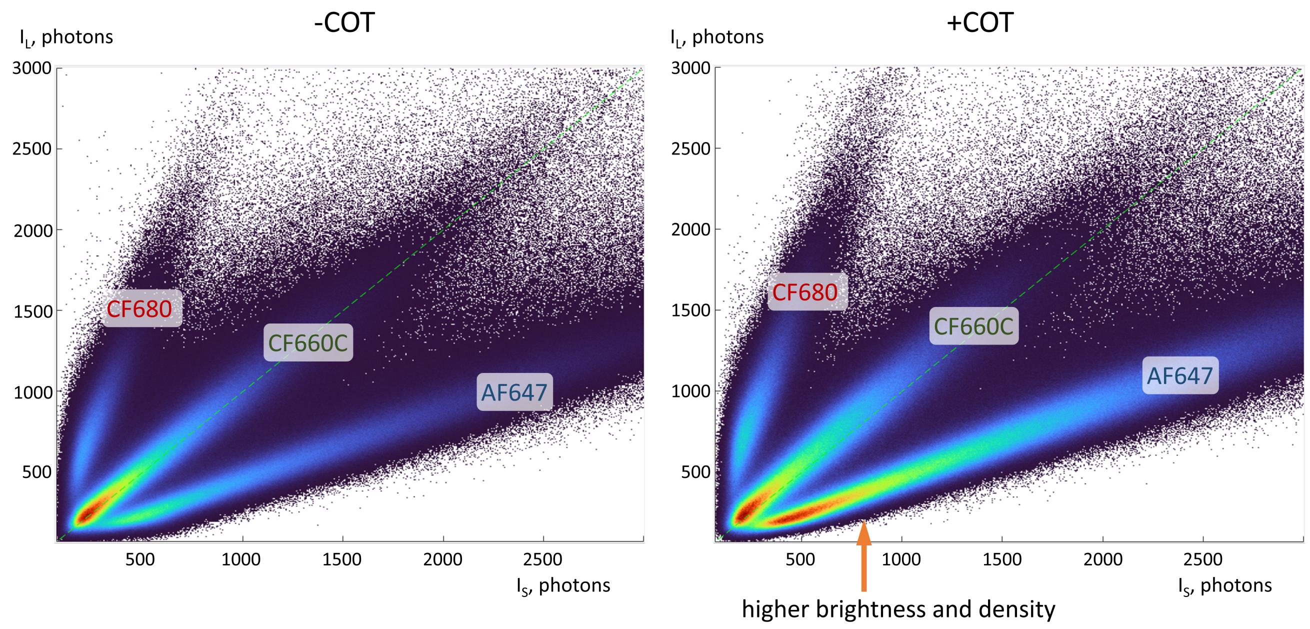

Suppl. Fig. S15 Effect of COT on the bivariate histogram of photon counts

Bivariate histogram of photon counts of a triple-labelled sample in an imaging buffer supplemented with 2 mM COT (right) indicates improved brightness and density of fluorophores as compared to a buffer without COT (left).

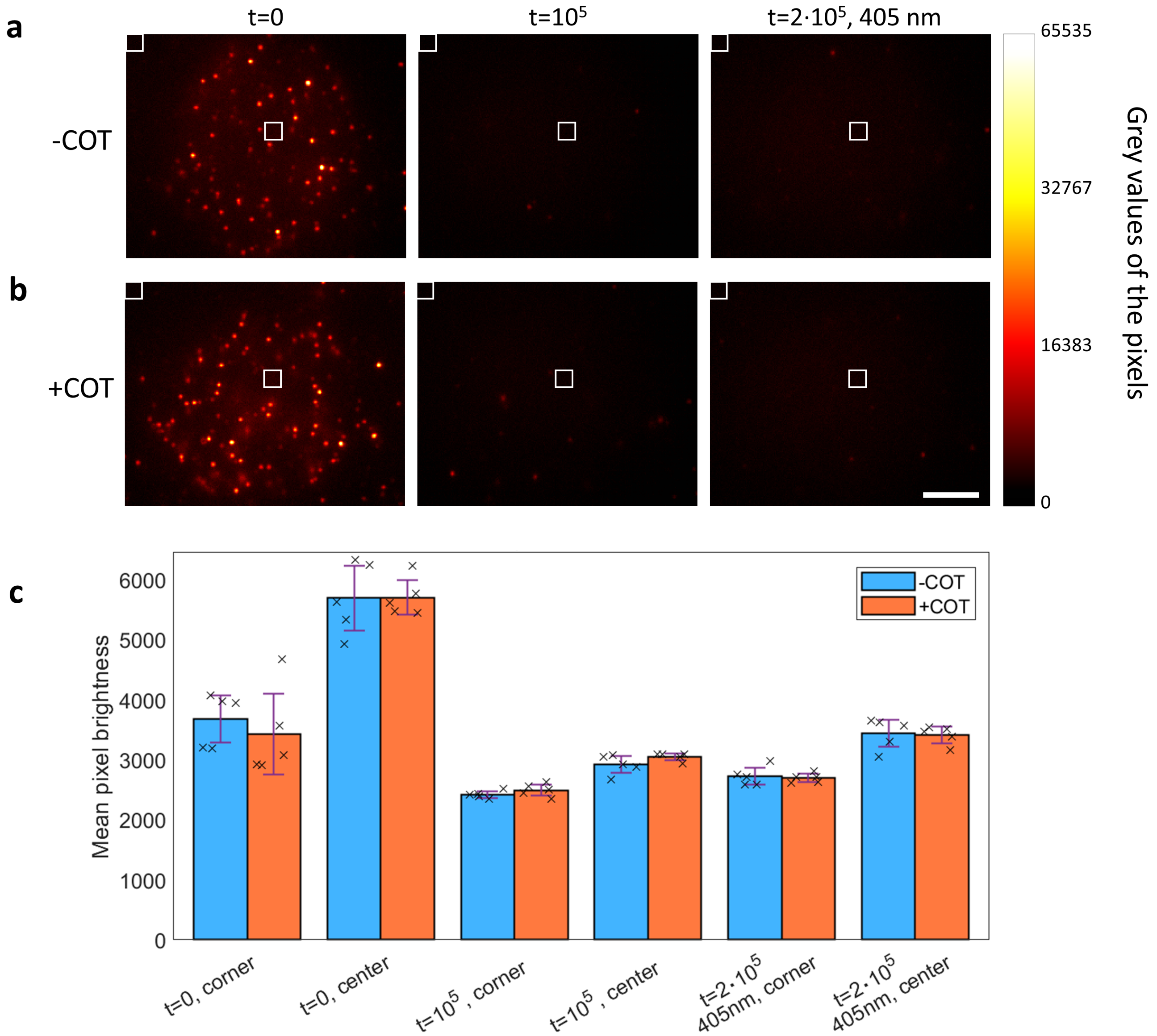

Suppl. Fig. S16 Quantification of autofluorescence of COT

(**A-B**) Images of an AF647-labelled U2OS Nup96-GFP cell at different time points in a Glox buffer without (**A**) or with COT (**B**), where the square regions used for autofluorescence quantification are indicated; scale bar, 5 µm. (**C**) Statistics on the signal within the square regions at different time points. The error bars indicate the mean ± standard deviation values after analysis of five different cells. The time *t* represents the frame index, where 0 corresponds to the first frame of the acquisition, $1.01 \cdot 10^5$ to the last frame of an acquisition without 405 nm-induced reactivation and $2 \cdot 10^5$ to the last frame of acquisition with 100% power of the 405 nm laser. The 642 nm laser intensity was kept at 40% throughout the whole experiment.

Supplementary Methods

Installation instructions

SplitViSu can be used as a separate stand-alone application for Windows or as a part of the SharpViSu package.

Download the latest version of the installer under https://github.com/andronovl/SharpViSu/tree/master/Installer

The installer will determine if the appropriate version of the MATLAB Compiler Runtime is already installed on the computer. If not already installed, the MATLAB Compiler Runtime will be downloaded and installed automatically. After the installation, run either SharpViSu.exe or SplitViSu.exe.

The software can be also used as a MATLAB application. Needs MATLAB R2021b or newer with Image Processing Toolbox, Signal Processing Toolbox, Statistics and Machine Learning Toolbox. This option allows to run the software also under Linux or Mac. For installation, download https://github.com/andronovl/SharpViSu/tree/master/SharpViSu and add all the files to the MATLAB search path. Then, run either SharpViSu_App.mlapp or SplitViSu.mlapp.
